# Menstrual Cycle: The Importance of Both the Phases and the Transitions Between Phases on Training and Performance

**DOI:** 10.1007/s40279-022-01691-2

**Published:** 2022-04-29

**Authors:** Georgie Bruinvels, Anthony C. Hackney, Charles R. Pedlar

**Affiliations:** 1 St Mary’s University, Twickenham, London, UK; 2 Orreco Ltd, Business Innovation Unit, NUI Galway, Galway, Ireland; 3 Institute of Sport, Exercise and Health, UCL, 170 Tottenham Court Road, London, W1T 7HA UK; 4 University of North Carolina, Chapel Hill, NC USA

## Abstract

The authors present opinions based on their applied experiences of working with female athletes in combination with the existing evidence-based literature. Most of the existing menstrual cycle research focuses on a few steady-state time points within the pre-defined menstrual cycle phases, yet this disregards the day-to-day hormonal changes that women have to accommodate to perform optimally and consistently. The traditional research models are inadequate for studying symptoms and symptom management, and ultimately for supporting athletes to perform well throughout the entirety of their cycle. As such, the monitoring of the day-to-day variation, particularly during the transitions between menstrual cycle phases appears to be an important “overlooked” consideration. This is particularly pertinent considering the known intra-individual and inter-individual variation in menstrual cycle characteristics. Anecdotal and research evidence supports the idea that athletes can use non-pharmacological solutions to mitigate negative menstrual cycle symptoms and do not need to “grit their teeth and roll with it”. However, further research (including case studies) is needed in this important research area. Such knowledge should be and needs to be widespread amongst practitioners and athletes as they should not have to figure this out alone. As such, researchers and practitioners need to put more work into understanding symptom aetiology, symptom clusters and their relationship with hormonal changes, menstrual cycle phases and transitions, with potential for a profound impact on individual athlete health and well-being. In so doing, those working with female athletes need to continue building on the recent progress made in educating athletes and practitioners; for example, normalising the discussion of and about the menstrual cycle and all of its implications.

## Background

Female sex hormone levels in eumenorrheic women can change by over 100% in a 24-h window of time [1]. While the physiological and psychological implications of such dramatic changes are incompletely understood, our first-hand experience with athletes over many years tells us that it is these sharp alterations in sex hormone levels that can be associated with adverse, often detrimental, symptoms. Common symptoms such as severe cramps, vomiting and injury flare-ups can all be debilitating and wholly incompatible with peak performance. Therefore, it is critical that the significance of these occurrences in the sport setting not be overlooked if practitioners are to provide helpful support to female athletes.

Our intention in this commentary is to assert that there is a clear case for practitioners and researchers to: (1) recognise the impact that menstrual cycle (MC) symptoms can have on aspects of performance and overall wellness (e.g. sleep patterns, recovery time and mood) throughout the entirety of the MC and (2) to not have an over-simplifying perspective on the MC phases (i.e. just mid-follicular vs mid-luteal), and in turn raise awareness of the importance of the day-to-day variation including transition periods between MC phases on athletes training and performance.

## MC Model

In the literature, the MC has frequently been split into physiological phases from the most simplistic (two phases) to the highly detailed (seven phases) [6]. However, most of the prior research informing our practices with athletes tends to rely on a three-phased model with the assumption of steady-state hormone levels existing; menstruation (low sex hormones, early follicular), pre-ovulation (high oestrogen, late follicular) and luteal (high oestrogen and progesterone). Yet this three-phased model disregards the hormonal changes punctuating the transitions from oestrogen dominance (late follicular phase) to ovulation, then to a greater hormone level phase where both oestrogen and progesterone are prominent (luteal phase), and finally returning to menstruation. The translational utility of these mid-phase studies for athletic use is weak. The hormonal changes during phase transition can be rapid and large in magnitude creating a dramatic change in the hormonal milieu of a woman and challenging the maintenance of homeostasis [1]. Crucially, the pre-menstrual phase, typically bracketed into the luteal phase, is a key window of significant hormonal decline and has been almost exclusively ignored in its own right within sports science research. Yet, it is in this pre-menstruation phase and during the subsequent early follicular phase (menstruation) where we have observed adverse MC symptoms in athletes to be most prevalent, and where there is an increased likelihood of a need for extended exercise recovery [7], compromises to training and performance [8]. This pre-menstruation window seems little studied or understood by sports science researchers, thus there is minimal guidance for practitioners. Similarly, the post-ovulatory increase in progesterone can also challenge systemic physiology. Progesterone has numerous systemic actions on other tissues, including affecting neuromodulation, metabolism and thermoregulation and increasing basal body temperature as well as having antiestrogenic actions [9–11]. Athletes need to be able to perform consistently throughout their MC, thus sports scientists must not be remiss and apply a reductionist approach to research to support both practitioners and athletes in maintaining consistent performance throughout the entire hormonal cycle.

## Symptom Burden

Besides anecdotal reports from athletes, evidence-based findings support a physiological basis for MC symptoms. One primary mechanism involves the pre-menstrual progesterone withdrawal and associated acute-phase inflammatory response [2]. This, when occurring in parallel with an intense training load (or other causes of stress both psychological and physical) has the potential to create the ‘perfect storm’ of excess fatigue and underperformance in athletes, if not managed proactively [3]. That said, not all women and their cycles are alike, and it is important to appreciate the significant inter-individual and sometimes intra-individual variation in cycle length, bleeding pattern, symptom type, severity and timing [4, 5]. The magnitude of symptoms, i.e. the number, frequency and severity; and the common clusters of symptoms have yet to be fully documented, but anecdotally, athletes can report feeling great one day, and compromised by symptoms just 1 day later. In our experience, it is rare to find female athletes with no negative symptoms whatsoever across their MC, but we recognise this is not a universal occurrence. Despite this, empirical evidence evaluating objective measures of performance (not symptoms) from cohort studies at fixed, steady-state MC time points are inconclusive with regard to whether there are performance impacts [6]. This ambiguity in the existing evidence is a call for more research.

## Global Consideration for Exercising Women

When considered en masse, over 90% of eumenorrheic (regularly menstruating) exercising women experience MC-related symptoms (e.g. changes in mood, menstrual cramps, fatigue, lower back pain), with 80% reporting symptoms or interrelated performance decrements every cycle [5, 8]. Symptoms appear to be as common in the general population and in elite athletes, and specific to the latter, recent findings suggest 50–67% of elite athletes perceive their exercise performance to be disrupted by their MC [8, 12, 13]. Furthermore, beyond the elite arena, given that adverse symptoms are a known barrier to habitual exercise, better understanding these symptoms, and subsequently finding suitable ways of mitigating them, could positively impact the lives of millions of women worldwide. The current situation is a wonderful opportunity for translational research. Historically, a lack of education, knowledge and willingness to discuss this topic has been a substantial limiting factor, but fortunately, this is rapidly changing.

## What Needs to be Done? Actions!

The first priority is to continue to educate both athletes and practitioners to normalise the conversation around the MC and raise awareness that symptoms (negative or positive) are common and are related to the natural physiological rhythms (i.e. hormonal changes) of the cycle. In addition to this, we advocate cycle tracking, and symptom logging to understand each individual’s profile (and to be able to detect deviations). Cycle tracking can take a number of different forms, including a simple paper diary or alternatively, there are several mobile applications with cycle tracking features. In full disclosure, two authors (GB, CP) of this article must acknowledge their involvement in the creation of the FitrWoman mobile application, and that while this is a tool that can be used in this instance, there are several other options.

Importantly, in our experience, negative symptoms can often be mitigated with appropriate multi-disciplinary non-pharmacological interventions and via trial and error (see following), helping athletes to avoid routine use of over-the-counter analgesics and anti-inflammatory drugs (e.g., non-steroidal anti-inflammatory drugs) or hormonal contraception as a primary treatment option, which can each present alternative concerns. Additionally, where resources are available, measuring hormones regularly could be informative, helping to specifically identify MC phases, and the magnitude or absence of hormone fluctuations (note: hormone *receptors* are also expressed differently over a MC [14], which may limit the insights that can be gained from just measuring circulating hormones alone [15]). Ultimately of course, as practitioners we need to be treating and managing the symptoms, not just the hormone levels. We propose that the key element here is how an individual responds to the changing ratio of reproductive hormones that present dynamically throughout the cycle; therefore, monitoring the dates of the cycle transients together with symptoms can provide vital practical information and preclude the essentiality, inconvenience and expense of regular hormone profiling, which most likely is not available to the non-elite female exerciser, and seldom available to the elite athlete. Furthermore, the language used when educating and discussing the MC should be carefully considered. The societal constructs associated with negative unwanted symptoms before and during menstruation need to be reframed to avoid artificially creating or reinforcing a barrier. Put simply, education should be used to enable and empower, not limit.

Like many strategies adopted by athletes, there might not be randomised controlled research trials providing evidence to support every intervention strategy in every scenario, and there probably never will be. However, there is evidence that supports nutritional interventions (e.g. curcumin and omega-3 intake) [16, 17] and exercise modes [18]. Further, use of particular nutritional interventions and extending sleep can reduce inflammation, which appears to be a key factor in negative symptom development [19–23]. Unfortunately, as far as we know, all such studies thus far demonstrating anti-inflammatory effects of nutritional interventions have been conducted exclusively in men. Nonetheless, we have observationally noted that these strategies can have a profound impact on women too. The advantage of nutrition and sleep interventions is that they do no harm, in contrast to the potential risks associated with long-term use of non-steroidal anti-inflammatory drugs or counteractive hormonal contraceptive use. Despite what is proposed herein, we acknowledge our current insights are based upon a paltry amount of evidence, further high-quality research is needed to provide data-driven insights into symptoms (frequency and severity), cycle length changes and symptom clusters as well as testing non-pharmacological, MC phase-appropriate, symptom mitigation strategies throughout the entirety of the MC.

## Conclusions

In female athletes, there is the potential and thus an overt need to perform on any day of the MC, consequently the impact of the day-to-day fluctuations in MC hormones and symptoms needs to be better understood (i.e. studied, monitored). Such monitoring in turn should be accompanied by education and, where possible and needed, individualised proactive management. Finally, the apparent disconnect between much of the existing research and applied practice needs to be addressed and rectified if we are to optimise the potential of female athletes.
